# Aseptic meningitis caused by torque teno virus in an infant: a case report

**DOI:** 10.1186/s13256-019-2233-2

**Published:** 2019-09-24

**Authors:** Yoji Ikuta, Kunihiro Oba, Emina Nai, Tatsuo Katori, Masanori Hashino, Yuba Inamine, Satoko Matsunaga, Yutaro Yamaoka, Tsuyoshi Sekizuka, Akihide Ryo, Makoto Kuroda

**Affiliations:** 1 Department of Pediatrics, Showa General Hospital, 8-1-1 Hanakoganei, Kodaira, Tokyo 187-8510 Japan; 2 Pathogen Genomics Center, National Institute of Infectious Diseases, 1-23-1 Toyama, Shinjuku, Tokyo 162-8640 Japan; 3 Yokohama City University Graduate School of Medicine, 1-7-29 Suehiro-cho, Tsurumi-ku, Yokohama 230-0045 Japan

**Keywords:** Torque teno virus, TTV, Aseptic meningitis, Infant, Next generation sequencing

## Abstract

**Background:**

Torque teno virus-induced aseptic meningitis has not been documented, although torque teno virus infections still remain under consideration for etiological agents. This study identified a torque teno virus sequence using next generation sequencing and immunoglobulin M response to the torque teno virus antigen, therefore, that would be a comprehensive diagnosis for torque teno virus infection.

**Case presentation:**

A 2-month-old Japanese boy was brought to our hospital because he was irritable, drowsy, and lethargic. He was admitted based on his test results which indicated the possibility of septic meningitis. He was started on treatment with high-dose antibiotics and steroids. On the third day of hospitalization, he became afebrile with improvement in his general status and was discharged on the sixth day. He had no developmental problems for up to 1 year after discharge. Metagenomic ribonucleic acid-Seq pathogen detection using next generation sequencing of a sample of his cerebrospinal fluid, which was collected at admission, revealed three short reads homologous to those in torque teno virus out of a total of 1,708,516 reads. This finding indicated that our patient was positive compared to the torque teno virus-negative cerebrospinal fluid samples (controls) from 13 other patients. The torque teno virus has been shown to have a whole genome sequence of 2810 nt by polymerase chain reaction. We prepared a recombinant GP2 antigen from torque teno virus and used it to study our patient’s anti-torque teno virus immune response. An anti-GP2 serum immunoglobulin M response was detected, providing further supportive evidence of torque teno virus infection.

**Conclusions:**

This case speculates that torque teno virus-induced aseptic meningitis has a good course. New technologies like next generation sequencing can help in the identification of such cases, and an accumulation of future cases is expected.

## Background

Torque teno viruses (TTVs) are ubiquitous viruses which are highly prevalent in several mammalian species and are currently classified into the family *Circoviridae* and genus *Anellovirus*. TTV has a single-stranded circular DNA of approximately 3.8 kb. Human TTVs were first reported in 1997 [1] and are epidemiologically associated with several human diseases such as respiratory illnesses, autoimmune disorders, and hepatitis. However, due to lack of sufficient information regarding their pathogenesis and specific disease etiologies, their direct involvement in these diseases has been debated [2]. Here we report a case of aseptic meningitis associated with TTV.

## Case presentation

A 2-month-old Japanese boy (KS025) was brought to our hospital due to irritability, drowsiness, and lethargy. He had a cough for approximately 1 week and a fever for 3 days before admission. He was admitted because he was very pale, and the results of an examination showed the possibility of septic meningitis.

He was born at 39 weeks, and his birth weight was 3788 g. At birth, he was admitted to the neonatal intensive care unit for 6 days due to transient tachypnea of the newborn. He had received one immunization each against *Haemophilus influenzae* type b (Hib), pneumococcal conjugate vaccine (PCV), *Rotavirus* (Rota), and hepatitis B virus (HBV) before admission. His brother and father also had a cough for 2 weeks. He had no relevant family history.

His vital signs at admission were as follows: body temperature, 37.5 °C; pulse rate, 156 beats per minute (bpm); respiratory rate, 30 breaths/minute; and blood oxygen saturation (SpO_2_), 98%. His body height was 65.0 cm and body weight was 5992 g. On physical examination, he appeared irritable and pale. His anterior fontanelle was not bulging, and there were no murmurs, rales, abdominal abnormal features, or exanthemas. The capillary refilling time was within 2 seconds.

A blood examination revealed a slightly elevated level of C-reactive protein (CRP) and increased white blood cell (WBC) count. However, urine WBC levels were normal, and a chest radiograph showed no evidence of pneumonia. Cerebrospinal fluid (CSF) was remarkable for neutrophilic pleocytosis, although protein and glucose levels were within normal ranges (Table 1). A brain computed tomogram revealed no abnormalities.

**Table 1:** Summary of the laboratory data for Patient KS025

Chemistry	
Na	135 mEq/L
K	4.9 mEq/L
Cl	100 mEq/L
BUN	8 mg/dL
Creatinine	0.24 mg/dL
CRP	0.76 mg/dL
Blood gas analysis	
pH	7.453
pCO_2_	29.1 mmHg
HCO_3_-	20.1 mmol/L
Base excess	−2.6 mmol/L
Glucose	112 mg/dL
Lactate	3.7 mmol/L
CBC	
Hemoglobin	9.9 g/dL
Platelet	48.8 × 10^4^/μL
WBC	13,900/μL
Neutrophil	50%
Eosinophil	1%
Monocyte	6%
Lymphocyte	43%
CSF	
Cell	673/μL
Neutrophil	91%
Lymphocyte	8%
Monocyte	1%
Protein	48 mg/dL
Glucose	63 mg/dL
Cl	123 mEq/L

*BUN* blood urea nitrogen, *CBC* complete blood count, *CRP* C-reactive protein, *CSF* cerebrospinal fluid, partial pressure of carbon dioxide, *pCO*_*2*_ partial pressure of carbon dioxide, *WBC* white blood cell

Because he was suspected of having septic meningitis because of above observations, he was given ampicillin (300 mg/kg per day), cefotaxime (300 mg/kg per day), and dexamethasone (0.15 mg four times a day). He became afebrile on the third day of hospitalization, and his general status improved. The antibiotic and steroid therapies were discontinued on the fourth day and, since his condition was stable, he was discharged on the sixth day. Bacterial culture tests were negative from two samples each of blood, urine, and CSF.

**Fig. 1:**
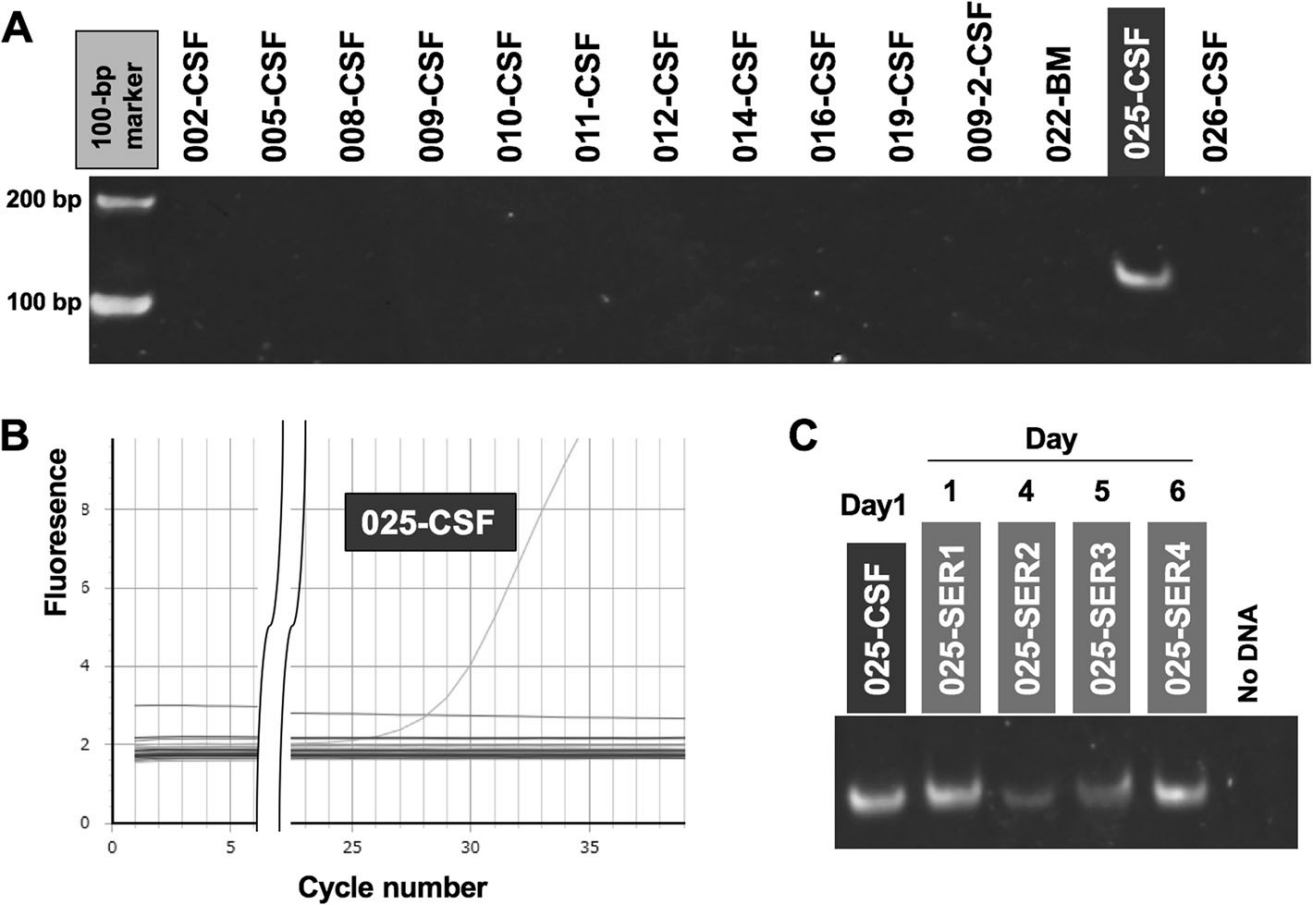
Torque teno virus detection using polymerase chain reaction. **a** Polymerase chain reaction detection using torque teno virus (KS025-cerebrospinal fluid)-specific primers in the cerebrospinal fluid and bone marrow. For comparison, 13 case controls were used. **b** Testing for torque teno virus-specific primers (KS025-cerebrospinal fluid: Ct 28.0) using quantitative reverse transcription-polymerase chain reaction. **c** Torque teno virus detection in the sequential serum samples of KS025. *BM* bone marrow, *CSF* cerebrospinal fluid, *SER* sequential serum

After discharge using a sample of our patient’s CSF which was collected at admission, we performed metagenomic RNA(ribonucleic acid)-Seq pathogen detection by next generation sequencing (NGS) [3]. Out of a total of 1,708,516 reads, three short reads homologous to those seen in TTV were detected. Next, we used our patient’s TTV-specific polymerase chain reaction (PCR) primers (5′–GGTTTCAGGTAGGTAGACA–3′ and 5′–GCCGAAGGTGAGTGAAA–3′) for quantitative PCR (qPCR) using TaKaRa SYBR® Fast qPCR Mix. We found that he was positive when compared with TTV-negative CSF samples from 13 control cases (Fig. 1). Whole genome sequence of TTV has been determined to be of 2810 nt by PCR amplification followed by NGS and has been deposited in the DNA Data Bank of Japan (DDBJ; Acc. no: LC381845).

**Fig. 2:**
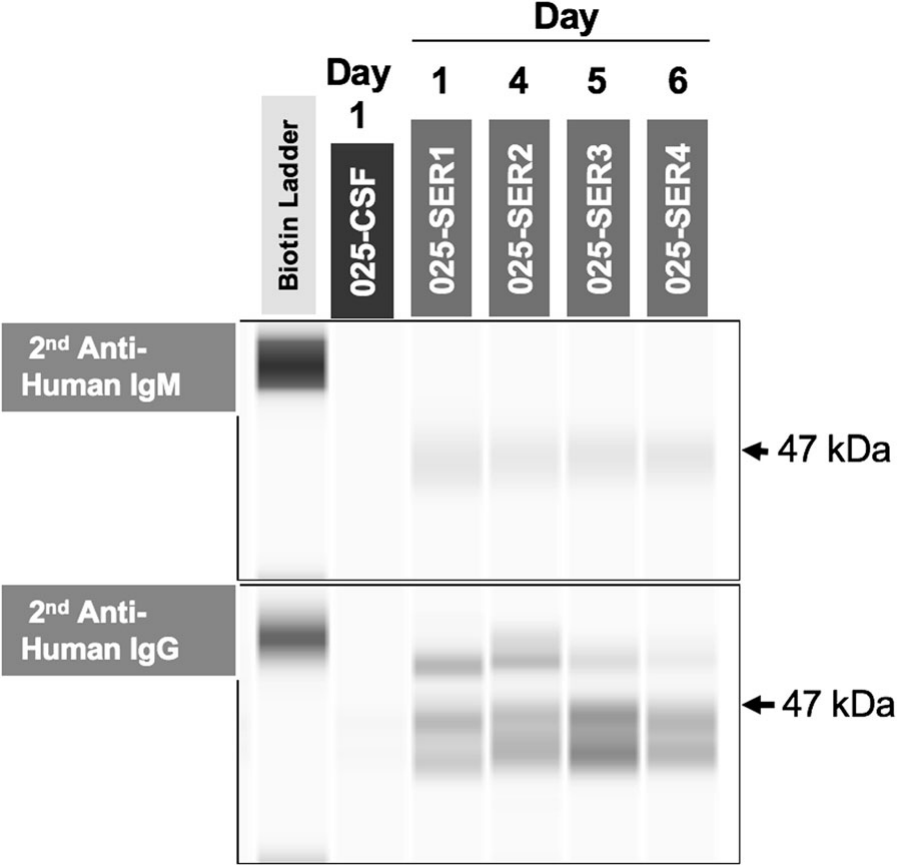
Immune detection of torque teno virus using a recombinant GP2 antigen. The torque teno virus-induced immune response in patient KS025 was assessed using a recombinant GP2 antigen (47 kDa, 270–663 aa region in total: GenBank ID, BBD88555) by an automatic Western blot detection system, Wes. *CSF* cerebrospinal fluid, *SER* sequential serum

We prepared a His-tagged recombinant GP2 (270–663 aa region, GenBank ID: BBD88555) antigen from TTV using WEPRO7240H wheat germ extract and the Protemist® DT II robotic protein synthesizer (CellFree Sciences Co., Matsuyama, Japan). We tested our patient’s immune response to the specific GP2 antigen using the automatic Western blot detection system Wes (ProteinSimple, San Jose, CA, USA) (Fig. 2). Neither IgM (immunoglobulin M) nor IgG (immunoglobulin G) was detected in the CSF, although serum was positive for both. Basically, serum IgG is received from the mother, thus we could not conclude the immune response to the TTV infection; however, the serum IgM response observed in this patient provided supportive evidence for TTV infection.

We followed up our patient for 1 year after discharge. He has had no developmental problems, can now stand by himself, and has started talking. He can also climb steps and pick up small items with his fingers. His mental and motor development are both progressing well.

## Discussion and conclusions

This is the first report in the world on TTV-associated aseptic meningitis. TTV was first reported in 1997 to be associated with elevated transaminase levels in post-transfusion hepatitis [1], followed by torque teno mini virus in 2000, and torque teno midi virus in 2007 [2]. Although these viruses have a high prevalence in the general population across the globe, their pathogenesis is not well understood. Although suggested to be associated with hepatitis, respiratory diseases, cancer, and hematological and autoimmune disorders [2], no cases of meningitis associated with TTVs have been reported yet.

In this case, the symptoms of respiratory infection, such as the cough, could not be related to TTV infection because sputum was not analyzed. The cough could have been a result of another preceding infection which could have also increased the permeability of the blood–brain barrier (BBB), thereby allowing the subsequent TTV infection to cause progressive neurological pathogenesis.

The relationship that TTV establishes with the central nervous system (CNS) of the infected hosts is not clear, but it is suspected to increase the local expression of inflammatory mediators that may play a role in the pathogenesis of multiple sclerosis [4]. It has been reported that analysis of specimens (bone marrow cells) from children with acute lymphoblastic leukemia were all found to be negative for TTV [5]. However, some patients turned out to be TTV positive later on, although TTV was not detected in the CSF [5]. It is, therefore, suggested that TTVs are able to invade the CSF only when the BBB is breached. During infancy, TTVs may invade the CSF due to the immaturity of the BBB. TTVs were not detected in cases of pediatric acute encephalitis, encephalopathy, or meningitis using NGS [6, 7]. Thus, a correlation between TTVs and infectious diseases of the CNS is not yet known.

Unlike cases in the past, using NGS we could detect TTV in this case of CNS infection. This case also shows that TTV-induced aseptic meningitis has a good course. New technologies like NGS can help in identifying cases of TTV as a causative virus, and we, therefore, expect an accumulation of such cases in the future.

## Data Availability

Whole genome sequence of TTV has been deposited in the DNA Data Bank of Japan (DDBJ; Acc. no: LC381845).
